# Tongue Color Analysis for Medical Application

**DOI:** 10.1155/2013/264742

**Published:** 2013-04-22

**Authors:** Bob Zhang, Xingzheng Wang, Jane You, David Zhang

**Affiliations:** ^1^Department of Electrical & Computer Engineering, Carnegie Mellon University, Pittsburgh, PA 15213, USA; ^2^Biometrics Research Center, Department of Computing, The Hong Kong Polytechnic University, Hong Kong

## Abstract

An in-depth systematic tongue color analysis system for medical applications is proposed. Using the tongue color gamut, tongue foreground pixels are first extracted and assigned to one of 12 colors representing this gamut. The ratio of each color for the entire image is calculated and forms a tongue color feature vector. Experimenting on a large dataset consisting of 143 Healthy and 902 Disease (13 groups of more than 10 samples and one miscellaneous group), a given tongue sample can be classified into one of these two classes with an average accuracy of 91.99%. Further testing showed that Disease samples can be split into three clusters, and within each cluster most if not all the illnesses are distinguished from one another. In total 11 illnesses have a classification rate greater than 70%. This demonstrates a relationship between the state of the human body and its tongue color.

## 1. Introduction

The human tongue contains numerous features that can be used to diagnose disease, with color features being the most important [1–4]. Traditionally, medical practitioners would examine these color features based on years of experience [5–11]. However, ambiguity and subjectivity are always accompanied with their diagnostic result. To remove these qualitative aspects, tongue color analysis can be objectively analyzed through its color features, which offers a new way to diagnose disease, one that minimizes the physical harm inflicted to patients (compared with other medical examinations).

A literature review on this topic revealed only a few papers where color features from the tongue are the main component used to diagnose disease. Reference [12] used tongue color along with qualitative and quantitative analysis to examine 207 patients suffering from lung cancer. The patients were split into four syndrome groups according to Chinese Medicine, and the CIELAB color model was used for quantitative classification. They reported significant statistical difference between the four groups when it came to each tongue's CIELAB value. The correct classification rate of each group was 69.4%, 54.4%, 72.2%, and 54.4%, respectively. A color metrics was utilized in [13] to diagnose appendicitis using tongue images. 798 tongue samples were captured from 399 patients (two samples from each person), consisting of common illnesses such as diabetes, pancreatitis, and hypertension, along with 114 images from tongues affected by appendicitis. The samples were captured using a specially designed device consisting of a 3-CCD digital camera, two D65 lights and calibrated with a white color plate. Four color spaces (RGB, CIE*xy*Y, CIELUV, and CIELAB) were evaluated to find the best combination. In their work they reported a correct classification of appendicitis to be 66.67%. Pancreatitis (29 samples) and appendicitis (53 samples) tongue images again appeared in [14], albeit this time with 56 normal samples. These images were captured with a device designed by their research center consisting of a lens, CCD sensor board, two D65 lights, and video frame grabber. Assessing the same four spaces as [13], the experimental results showed that normal and pancreatitis as well as appendicitis and pancreatitis can be linearly separated using color features.

In [12] the dataset was quite small and only one disease was analyzed. The patients were also diagnosed using Chinese Medicine. As for [13] its dataset was substantially larger but only appendicitis was classified. The samples in [14] include two illnesses as well as normal, but their sizes are too small to have any statistical significance. Both works in [12, 14] did not use any image correction to ensure uniform feature extraction and analysis under different operating conditions. Also, [12–14] used some variation of the CIE color space to embody the tongue colors, which may not be very accurate. Therefore, there is a lack of any work on an in-depth systematic tongue color analysis system for medical applications, one that accurately captures the images and represents its colors using a tongue color gamut [15]. In this paper such an application is described to address these problems. Tongue images are first captured using a specifically designed device with image correction. Afterwards, the images are segmented [16] with the background removed and tongue foreground remaining. Color features from each pixel are then extracted and assigned to 1 of 12 colors symbolizing the tongue color gamut [15]. This produces the tongue color feature vector. Experimental results were carried out on a large scale dataset consisting of 143 Healthy and 902 Disease samples (diagnosed using Western Medicine) taken from Guangdong Provincial Hospital of Traditional Chinese Medicine, Guangdong, China. The Disease class was composed of 13 specific illnesses (with at least 10 samples in each group) and one sizeable miscellaneous group (made up of various illnesses). Classification was performed between the illnesses in addition to Healthy versus Disease.

The rest of this paper is organized as follows. An introduction to the tongue image acquisition device and dataset used is given in Section 2.  Section 3 summarizes the tongue color gamut and explains how color features are extracted using it. In Section 4 classification between the two classes of Healthy and Disease is performed. Following this, illnesses in the Disease class are classified. Finally, concluding remarks are made in Section 5.

## 2. Materials

**Figure 1:**
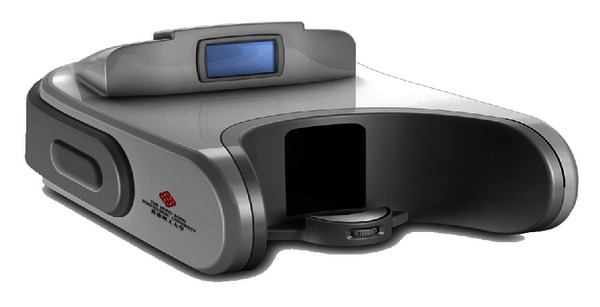
Tongue capture device.

The tongue database is composed of 1045 images (one image per person) split into 143 Healthy and 902 Disease captured at Guangdong Provincial Hospital of Traditional Chinese Medicine, Guangdong, China. The patients' consent was obtained according to the Declaration of Helsinki and the Ethical Committee of the Institution in which the work was performed approved it. The capture device used was a three-chip CCD camera with 8 bit resolution and two D65 fluorescent tubes placed symmetrically around the camera in order to produce a uniform illumination. The images captured were color corrected [17] to eliminate any noise caused by variations of illumination and device dependency. This allows consistent feature extraction and classification in the following steps.  Figure 1 shows the capture device. Healthy samples were verified through a blood test and other experiments. If indicators from the tests fall within a certain range they were deemed fit. In the Disease class, samples were collected from inpatients with illnesses determined by their admission note, diagnosed using Western Medical practices. Inpatients suffering from the same illness were grouped together into a single class. In total there were 13 ailment groups (with at least 10 samples) and one miscellaneous group containing various illnesses. A summary of the Disease class breakdown is given in Table 1. Please note any future reference to a specific illness in Table 1 will be made using its Disease ID.

## 3. Methods

The following section describes how color features are extracted from tongue images. The tongue color gamut is first summarized in Section 3.1. In Section 3.2, every foreground tongue pixel is compared to 12 colors representing the tongue color gamut and assigned its nearest color. This forms the color features. 

### 3.1. Tongue Color Gamut

**Figure 2:**
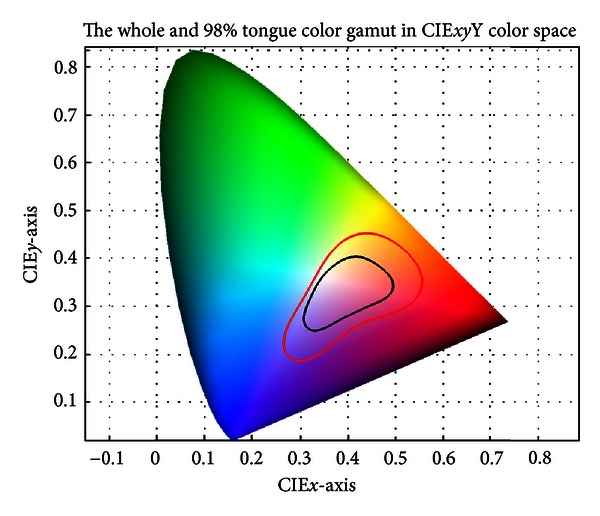
A color gamut in the CIE*xy*Y color space depicting the tongue color gamut inside the red boundary. Furthermore, 98% of the tongue color gamut can be located within the black boundary.

**Figure 3:**
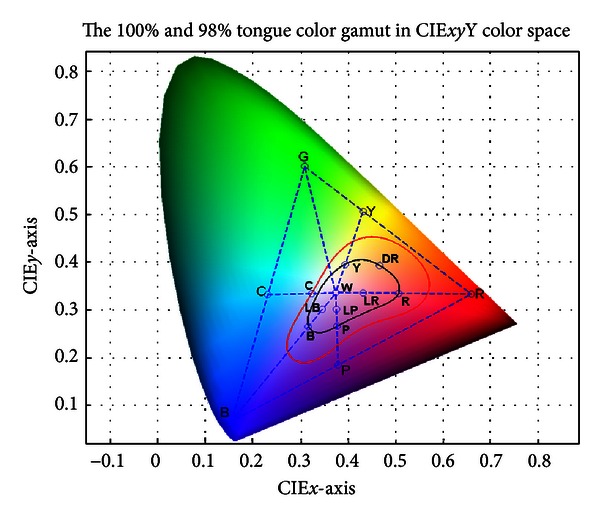
The tongue color gamut can be represented using several points by drawing lines from the RGB color space.

The tongue color gamut [15] represents all possible colors that appear on the tongue surface and exists within the red boundary shown in Figure 2 (CIE-*xy* chromaticity diagram). Further investigation revealed that 98% of the points lie inside the black boundary. To represent the tongue color gamut using 12 colors, the RGB color space is employed and plotted in Figure 3. On the RG line a point Y (Yellow) is marked. Between RB a point P (Purple) is marked and C (Cyan) is marked between GB. The center of the RGB color space is calculated and designated as W (White), the first of the 12 colors (see Figure 3). Then, for each R (Red), B (Blue), Y, P, and C point, a straight line is drawn to W. Each time these lines intersect the tongue color gamut, a new color is added to represent the 12 colors. This accounts for R, Y, C, B, and P. LR (Light red), LP (Light purple), and LB (Light blue) are midpoints between lines from the black boundary to W, while DR (Deep red) is selected as no previous point occupies that area. More details about the tongue color gamut can be found in [15]. GY (Gray) and BK (Black) are not shown in Figure 3 since both belong to grayscale.

**Figure 4:**
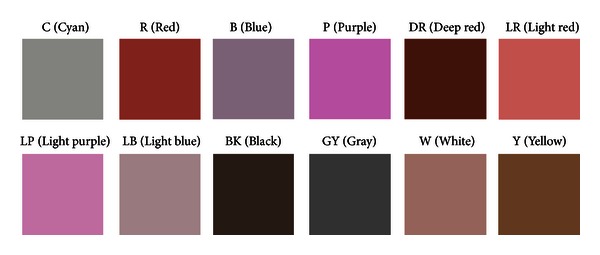
12 colors representing the tongue color gamut with its label on top.

The 12 colors representing the tongue color gamut are extracted from Figure 3 and shown in Figure 4 as a color square with its label on top. Correspondingly, its RGB and CIELAB values are given in Table 2.

### 3.2. Tongue Color Features

Given a tongue image, segmentation is first applied to locate all foreground tongue pixels [16]. Having located each pixel its corresponding RGB value is extracted and converted to CIELAB [18] by first converting RBG to CIE*XYZ* using

(1) $$\begin{array}{c} \left[\begin{array}{c} X\\ Y\\ Z \end{array}\right]=\left[\begin{array}{ccc} 0.4124 & 0.3576 & 0.1805\\ 0.2126 & 0.7152 & 0.0722\\ 0.0193 & 0.1192 & 0.9505 \end{array}\right]\left[\begin{array}{c} \text{R}\\ \text{G}\\ \text{B} \end{array}\right] \end{array}$$

followed by CIE*XYZ* to CIELAB via

(2) $$\begin{array}{c} L^{*}=116f\left(\frac{Y}{Y_{0}}\right)-16,\\ a^{*}=500\left[f\left(\frac{X}{X_{0}}\right)-f\left(\frac{Y}{Y_{0}}\right)\right],\\ b^{*}=200\left[f\left(\frac{Y}{Y_{0}}\right)-f\left(\frac{Z}{Z_{0}}\right)\right],\\ \text{where}\;\;f\left(x\right)=\{\begin{array}{cc} x^{1/3} & \left(x>0.008856\right),\\ 7.787x+\frac{16}{116} & \left(x\leq 0.008856\right). \end{array} \end{array}$$

**Figure 5:**
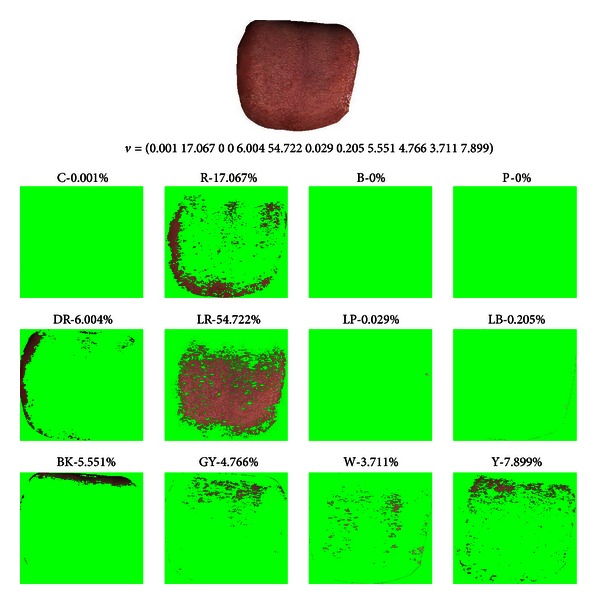
Healthy tongue sample, its tongue color feature vector and corresponding 12-color makeup with most of the pixels classified as LR.

**Figure 6:**
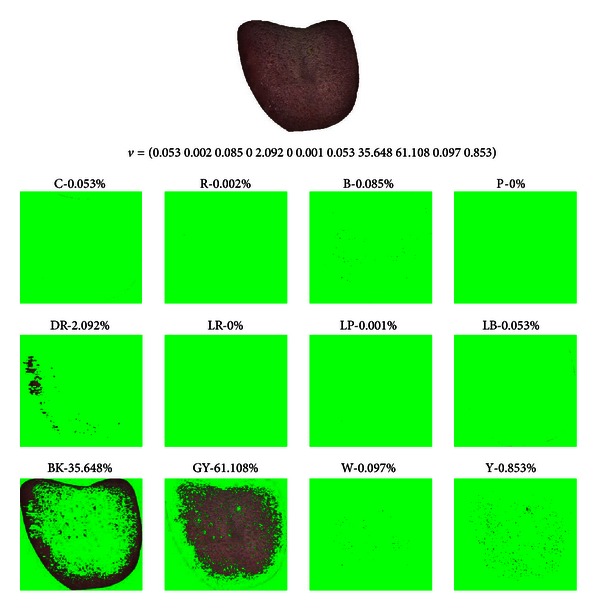
Disease tongue sample, its tongue color feature vector and corresponding 12-color makeup with most of the pixels classified as GY.

In (2), *X*_0_, *Y*_0_, and *Z*_0_ are the CIE*XYZ* tristimulus values of the reference white point. The LAB values are then compared to 12 colors from the tongue color gamut (see Table 2) and assigned the color which is closest to it (measured using Euclidean distance). After calculating all tongue foreground pixels, the total of each color is summed and divided by the number of pixels. This ratio of the 12 colors forms the tongue color feature vector *v*, where *v* = [*c*_1_, *c*_2_, *c*_3_, *c*_4_, *c*_5_, *c*_6_, *c*_7_, *c*_8_, *c*_9_, *c*_10_, *c*_11_, *c*_12_] and *c*_*i*_ represents the sequence of colors in Table 2. As an example, the color features of two tongues are shown in visual form (refer to Figures 5 and 6) along with its extracted tongue color feature vectors, where the original image is decomposed into one of the 12 colors.  Figure 5 is from a Healthy sample and Figure 6 is from a Disease sample. In the Healthy sample the majority of pixels are LR and for Disease it is GY.

**Figure 7:**
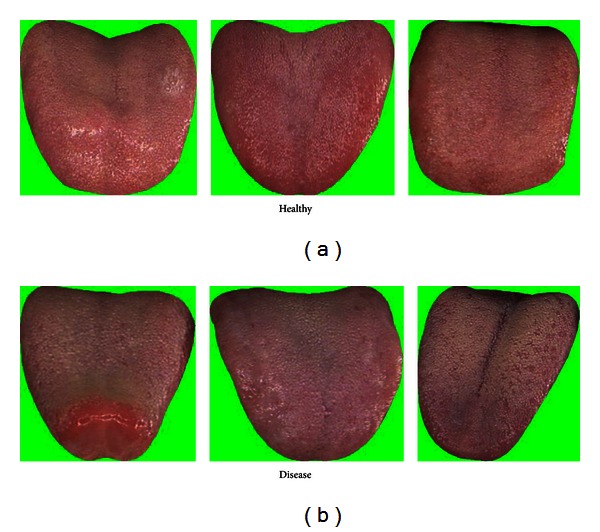
Three typical Healthy (a) and Disease (b) samples.

The mean colors of Healthy and Disease are displayed in Table 3 along with three typical samples from each class shown in Figure 7. Disease tongues have a higher ratio in R, DR, BK, GY, and Y according to Table 3. On the other hand, LR and W are greater in Healthy. Only 7 colors are listed out of the 12 as the remaining 5 have ratios less than 1%.

## 4. Results and Discussion

In this section classification using color features is described. Classification between Healthy versus Disease is first given in Section 4.1, while illnesses in Disease are classified in Section 4.2.

### 4.1. Healthy versus Disease Classification

Table 4 shows the classification rate between Healthy versus Disease on the test data. Half the images were randomly selected from either class to represent the training set and the remaining samples assigned to the test set. The training data in each class are the mean tongue color features of Healthy and Disease. To reduce the number of tongue color features, feature selection with sequential forward search was implemented. Both *k*-NN [19] and SVM [19] using a quadratic kernel were tested producing the same result as can be seen in Table 4. This means for *k*-NN and SVM the tongue color feature vector of the training set consisting of Healthy and Disease was placed in an *n*-dimensional space. Each tongue color feature vector representing the test set was mapped to this space and classified depending on its classification rule (*k*-NN or SVM).

### 4.2. Typical Disease Analysis

With Healthy versus Disease separated the next step is to examine whether certain illnesses within the Disease class can be distinguished from one another. All 13 illnesses were grouped into three clusters by FCM [19], with Table 5 illustrating which cluster each illness belongs to. The mean tongue color features of each cluster are shown in Table 6. R, DR, and LR are greater in Cluster 3. Cluster 2 has higher concentrations of GY, BK, and W, while Y is more significant in Cluster 1. 

**Figure 8:**
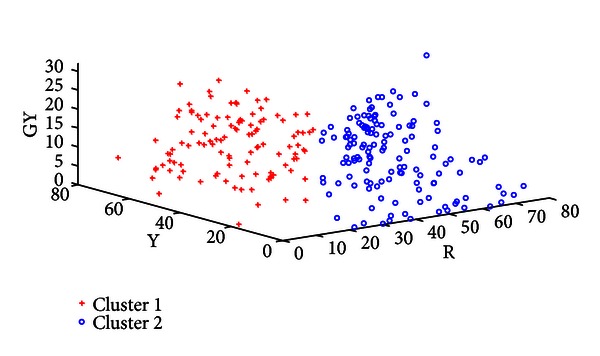
Plot of Cluster 1 versus Cluster 2.

**Figure 9:**
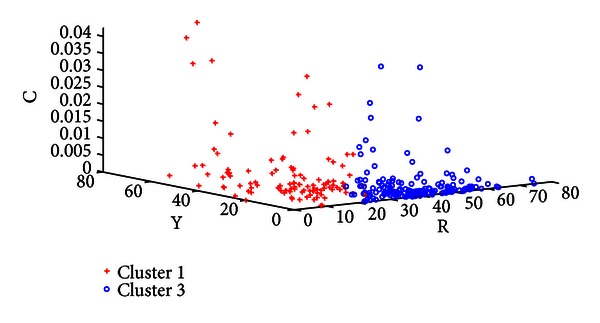
Plot of Cluster 1 versus Cluster 3.

**Figure 10:**
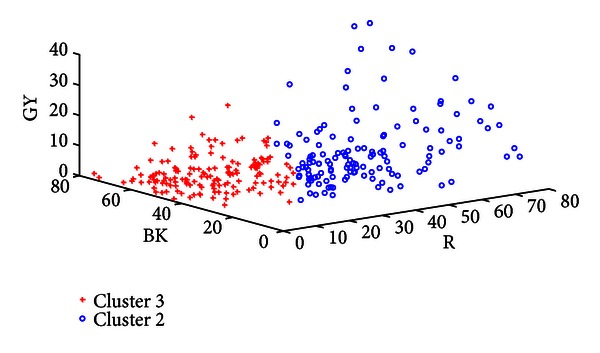
Plot of Cluster 3 versus Cluster 2.

**Figure 11:**
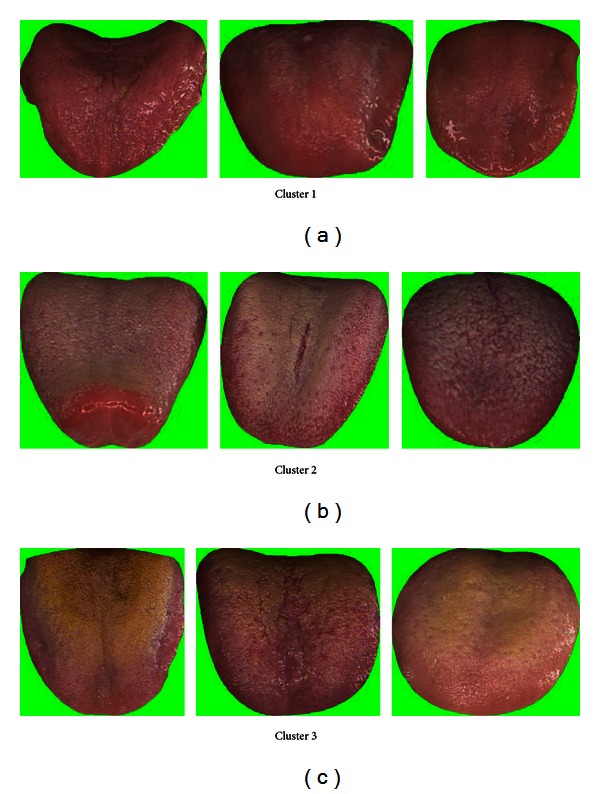
Three typical samples from each cluster.

Table 7 shows the classification rate of the three clusters calculated in groups of two. In each case the two clusters in question are clearly separable as seen in this table and Figures 8, 9, and 10. Three typical samples from each cluster are depicted in Figure 11. From a visual perspective the tongue color features in each cluster are quite different compared to the rest.

Next, each cluster is examined one by one to determine whether illnesses within it can be classified. This is accomplished by comparing illnesses inside the cluster and removing the illness with the highest classification. The process is repeated until all illnesses have been classified. The same experimental setup described in Section 4.1 was applied, where half the images are randomly selected for training and test sets. Both *k*-NN and SVM were used as the classifiers along with sequential forward search for feature selection. An illness is considered successfully classified if its average accuracy is greater than or equal to 70%. The average accuracies stated in the following paragraph represents only SVM. For a complete list of the results please refer to Table 8.

**Figure 12:**
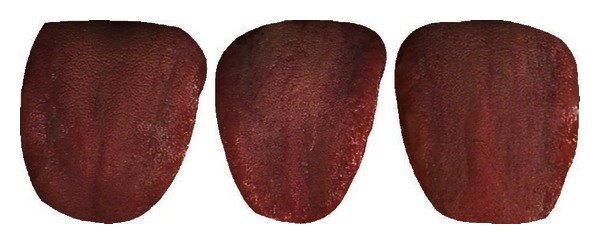
Three typical samples from Disease 1.

**Figure 13:**
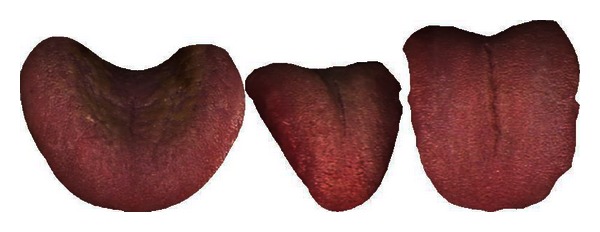
Three typical samples from Disease 3.

**Figure 14:**
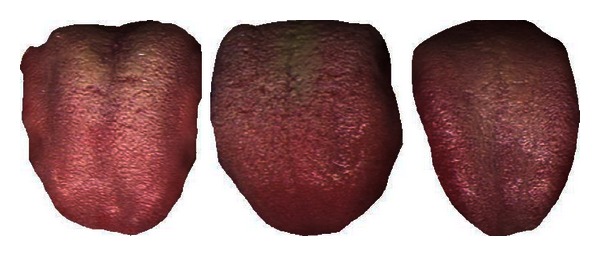
Three typical samples from Disease 5.

**Figure 15:**
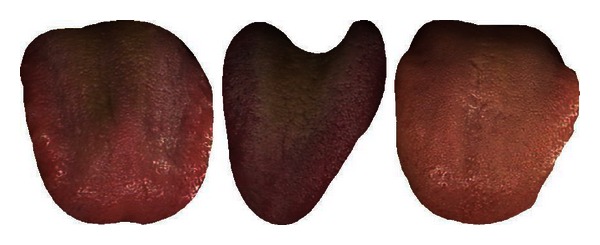
Three typical samples from Disease 6.

**Figure 16:**
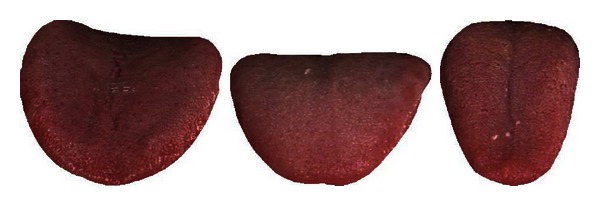
Three typical samples from Disease 7.

**Figure 17:**
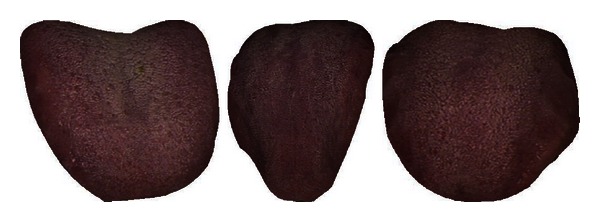
Three typical samples from Disease 8.

**Figure 18:**
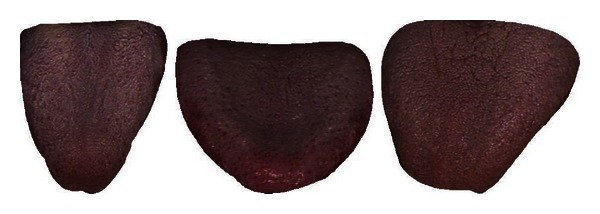
Three typical samples from Disease 9.

**Figure 19:**
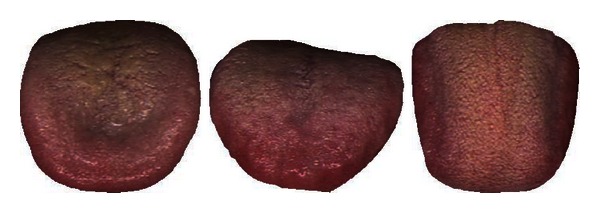
Three typical samples from Disease 10.

**Figure 20:**
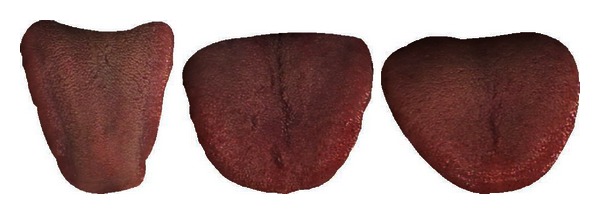
Three typical samples from Disease 11.

**Figure 21:**
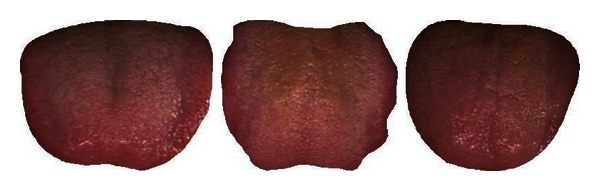
Three typical samples from Disease 12.

**Figure 22:**
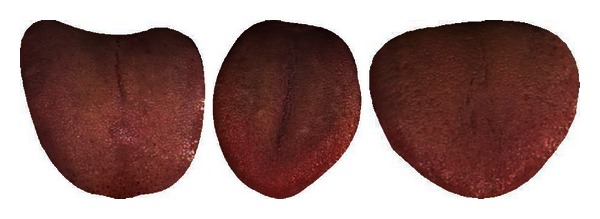
Three typical samples from Disease 13.

Diseases 1 and 13 in Cluster 1 are separable with an average accuracy of 76.08%. In Cluster 2, Disease 7 can be first removed as its classification rate of 93.06% is the highest amongst the six illnesses. Diseases 10, 8, and 9 are subsequently taken out in that order which leaves illnesses 5 and 12 (classification rate of 81.45%). Looking at Cluster 3, Disease 6 with a classification rate of 74.05% is initially removed from the pack. This is followed by Diseases 3 and 11 leaving 2 and 4 which produced the lowest classification result of 54.41%. Table 8 summarizes this result. Diseases 1, 3, 5, 6, 7, 8, 9, 10, 11, 12, and 13 achieved an average accuracy greater than 70% and therefore deemed successfully classified. Typical samples of the successfully classified illnesses are shown in Figures 12, 13, 14, 15, 16, 17, 18, 19, 20, 21, and 22.

As part of the future work we plan on returning to Guangdong Provincial Hospital of Traditional Chinese Medicine and collect more diseased tongue images. Color features (discussed in Section 3.2) will be extracted from these new images before combining it with the previous batch. The experimental results in the form of Healthy versus Disease classification and typical disease analysis will be recalculated in order to further validate its statistical accuracy.

## 5. Conclusion

Given a tongue image the tongue color analysis system is able to first distinguish Healthy versus Disease with an average accuracy of 91.99%. If the image is from Disease it is further assigned to one of three clusters. From these clusters 11 illnesses can be successfully classified given a classification rate of at least 70%. The proposed method uses a special capture device with image correction and extracts a tongue color feature vector from each image. This vector consists of 12 color ratios calculated with the tongue color gamut to better characterize each foreground tongue pixel. Testing was carried out on a large dataset collected from Guangdong, China, consisting of 143 Healthy and 902 Disease images (13 specific illnesses with at least 10 samples and a miscellaneous folder). The experimental results showed that there is a relationship between tongue color and the state of the human body, which can be used in medical applications to detect various illnesses. This can potentially lead to a new painless and efficient way to examine patients.

## Figures and Tables

**Table 1:** Disease class statistics listing the ID, name, and number of samples.

Disease ID	Disease name	No. of samples
1	Chronic Kidney Disease	141
2	Diabetes	69
3	Nephritis	10
4	Hypertension	66
5	Verrucous Gastritis	25
6	Pneumonia	10
7	Nephritic Syndrome	10
8	Chronic Cerebral Circulation Insufficiency	14
9	Upper Respiratory Tract Infection	12
10	Erosive Gastritis	10
11	Coronary Heart Disease	13
12	Chronic Bronchitis	11
13	Mixed Hemorrhoid	11
14	Miscellaneous	500

**Table 2:** RGB and CIELAB values of the 12 colors.

Color	[R G B]	[L A B]
C (Cyan)	[188 188 185]	[76.0693 −0.5580 1.3615]
R (Red)	[189 99 91]	[52.2540 34.8412 21.3002]
B (Blue)	[183 165 180]	[69.4695 9.5423 −5.4951]
P (Purple)	[226 142 214]	[69.4695 42.4732 −23.8880]
DR (Deep red)	[136 72 49]	[37.8424 24.5503 25.9396]
LR (Light red)	[227 150 147]	[69.4695 28.4947 13.3940]
LP (Light purple)	[225 173 207]	[76.0693 24.3246 −9.7749]
LB (Light blue)	[204 183 186]	[76.0693 7.8917 0.9885]
BK (Black)	[107 86 56]	[37.8424 3.9632 20.5874]
GY (Gray)	[163 146 143]	[61.6542 5.7160 3.7317]
W (White)	[200 167 160]	[70.9763 10.9843 8.2952]
Y (Yellow)	[166 129 93]	[56.3164 9.5539 24.4546]

**Table 3:** Mean of the color features for Healthy and Disease.

	R	DR	LR	BK	GY	W	Y
Healthy	20.9284	5.6679	33.8483	8.2356	14.5583	7.9166	8.0485
Disease	28.2901	15.5951	11.0277	15.4325	16.2247	2.4990	10.6382

**Table 4:** Classification result between Healthy versus Disease using *k*-NN and SVM.

Classification method	Average accuracy
*k*-NN	91.99%
SVM	91.99%

**Table 5:** Distribution of the illnesses within the clusters.

Cluster number	Disease group
1	1 13
2	5 7 8 9 10 12
3	2 3 4 6 11

**Table 6:** Mean tongue color features of the three clusters.

Cluster number	R	DR	LR	BK	GY	W	Y
1	21.561	13.972	12.265	12.535	9.524	3.703	26.191
2	17.116	11.980	9.437	15.574	34.733	4.111	6.539
3	40.736	15.396	15.872	8.232	10.770	1.668	7.015

**Table 7:** Classification result between the three clusters compared in groups of two.

Cluster comparison	Average accuracy
Cluster 1 versus Cluster 2	100%
Cluster 1 versus Cluster 3	97.75%
Cluster 2 versus Cluster 3	99.63%

**Table 8:** Classification result of the illnesses.

Disease ID	Cluster number	*k*-NN	SVM
1	1	72.53%	76.08%
2	3	54.87%	54.41%
3	3	72.97%	75.68%
4	3	54.87%	54.41%
5	2	81.45%	81.45%
6	3	78.61%	74.05%
7	2	83.33%	93.06%
8	2	72.02%	83.33%
9	2	77.78%	83.33%
10	2	78.71%	87.10%
11	3	72.82%	73.56%
12	2	81.45%	81.45%
13	1	72.53%	76.08%
